# Respiratory pathology in late preterm infants conceived by *in vitro* fertilization

**DOI:** 10.25122/jml-2022-0194

**Published:** 2022-08

**Authors:** Octaviana Cristea, Ramona Mohora, Leonard Nastase, Alexandra Diaconu, Silvia-Maria Stoicescu

**Affiliations:** 1 Departament of Obstetrics, Gynecology and Neonatology, Carol Davila University of Medicine and Pharmacy, Bucharest, Romania; 2 Departament of Neonatology, Alessandrescu -Rusescu National Institute of Mother and Child Health, Bucharest, Romania

**Keywords:** *in vitro* fertilization, late-preterm infants, respiratory distress syndrome, oxygen therapy

## Abstract

This study aimed to identify the incidence of *in vitro* fertilization (IVF) in late preterm infants and the presence of respiratory pathology in this premature category compared with those conceived naturally. This retrospective study was performed over 6 months, including newborns with a gestational age between 34–36 weeks and 6 days in the Department of Obstetrics, Gynecology and Neonatology, Alessandrescu-Rusescu National Institute of Mother and Child Health. The following variables were assessed: infants' gestational age, delivery mode, respiratory morbidity, and the need for respiratory support. During the mentioned period, 112 late preterm infants were born, out of whom 9.8% represented late preterm infants conceived by *in vitro* fertilization. The delivery mode of late preterm infants conceived by *in vitro* fertilization was exclusively by C-section (100%) compared to those conceived spontaneously (44.5%). 18.1% of IVF late preterm infants developed transient tachypnea of the newborn. In the non-IVF group, respiratory distress syndrome was present in 5.9% and transient tachypnea in 33.6% of cases. No IVF late preterm infant required hospitalization in neonatal intensive care for more than 3 days, compared to 19.8% of naturally conceived late preterm infants. Respiratory distress syndrome very seldom occurs in late preterm IVF infants due to prenatal prophylactic treatment with corticosteroids. Respiratory pathology is rarely present due to very careful monitoring during pregnancy, the presence of a neonatal team in the delivery room for possible resuscitation, and providing proper care according to the good state of health during the short, one-week hospitalization.

## INTRODUCTION

*In vitro* fertilization (IVF), a method of assisted reproduction techniques (ART), was perfected by the British Scientist Professor Robert Geoffrey Edwards, allowing on July 25^th^ 1978, in London, the birth of Louise Joy Brown, the first child conceived through this method. IVF is considered one of the greatest medical achievements of the 20^th^ century, “one of the fastest growing areas of medicine, having expanded far beyond the imaginations of those who pioneered the technique that led to the birth of Louise Brown” [1]. Robert Geoffrey Edwards was awarded the 2010 Nobel Prize in Medicine. IVF has been recording constant worldwide growth. There are over 8 million children conceived by IVF [2]. In Timișoara, Romania, the first IVF child was born in 1996 thanks to Academician Ioan Munteanu. International studies show that a pregnancy after IVF is more likely to be a multiple pregnancy or a pregnancy with a higher risk of preterm birth and congenital anomalies. More than that, it is well known that IVF pregnancy has higher perinatal mortality and morbidity. [3].

Preterm birth is defined as the birth of a child before 37 fulfilled weeks of gestation and is the leading cause of neonatal mortality [4]. New global estimates show that in 2014, approximately 14.8 million infants were born preterm (10.6% of all live births) globally [5]. The major factors responsible for the increase in preterm delivery are the multiple births resulting from ART, high rates of late preterm (LPT) newborns, and maternal comorbidities [6].

The British Medical Journal defines prematurity categories based on gestational age (GA) (weeks-w): extreme prematurity (<28w), severe prematurity (28–31w), great prematurity (32–33w), and late prematurity (34–36w). This classification was necessary because LPT represent 8–9% of the newborns and 74% of the preterm neonates, their incidence rising by 20% since 1990 [5]. Late-preterm newborns are not completely mature. The last six weeks of gestation represent a critical period for the development of the lungs and brain [7, 8].

The objective of this retrospective study was to identify the incidence of *in vitro* fertilization of late preterm newborns (IVF-LPT) and the presence of respiratory pathology in this category of preterm newborns compared with those conceived naturally (non-IVF newborns).

## Material and Methods

The retrospective study was performed in 2019 for 6 months at the Department of Obstetrics, Gynecology and Neonatology, Alessandrescu-Rusescu National Institute of Mother and Child Health, Bucharest, Romania. We included 112 IVF and naturally conceived infants admitted to our department. The following were monitored: infants' gestational age, delivery mode, respiratory morbidity, the need for respiratory support, and mortality. Data were collected from medical records and analyzed using IBM SPSS Statistics v20.0.0, performing descriptive statistics.

## Results

**Figure 1:**
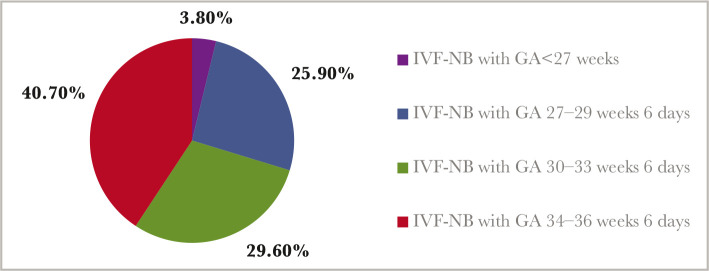
The distribution of IVF preterm infants by gestational age categories.

During the mentioned period, there were 44 IVF infants recorded, 61.4% of whom were born preterm. Late preterm infants (LPT) accounted for 25% of the total number of IVF infants and 40.7% of those who were born preterm (Figure 1).

**Figure 2:**
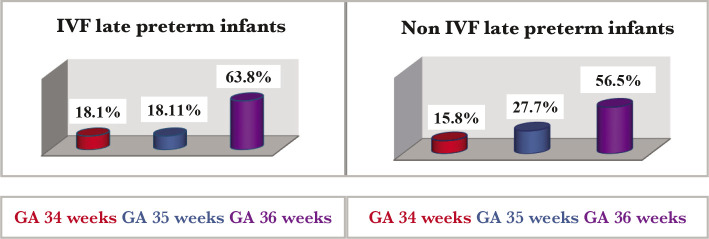
The incidence of IVF and non-IVF LPT according to gestational age.

During the study period, 112 preterm infants were born at a gestational age of 34–36 weeks and 6 days (LPT). LPT conceived by IVF accounted for 9.8% of the total number of LPT recorded during the study. According to gestational age, in both groups, LPT infants, in most cases, were born at 36 weeks of gestational age (Figure 2).

The incidence of IVF LPT newborns by gender was: female – 81.8%, male – 18.2%. In the non-IVF group, 51.4% were female, and 48% were male newborns. Regarding birth weight (BW), 54.5% of IVF-LPT infants had a BW over 2500 grams and 45.4% between 2000–2500 grams. In the non-IVF-LPT group: 52.4% had BW >2500 grams, 40.5% 2000–2500 grams, 5.2% between 1500–1999 grams and 1.9% were under 1500 grams. A big difference was registered regarding fetal presentation: the percentage of cranial presentation in IVF-LPT infants was 54.5% *vs*. 93.1% in the non-IVF group; the breech presentation was 45.5% in the IVF-LPT group *vs*. 6.9% in the non-IVF LPT infants.

**Figure 3:**
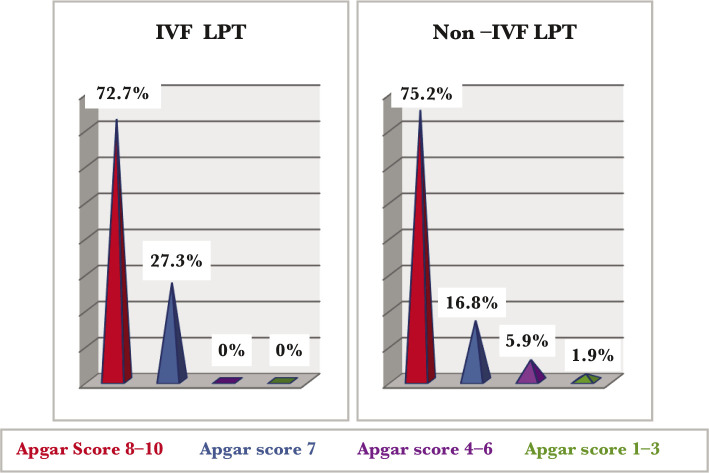
Perinatal asphyxia in IVF and non-IVF LPT.

There were differences in the Apgar score between the two groups: IVF-LPT infants had an Apgar score under 7, compared to the non-IVF group in which 5.9% had an Apgar score between 4–6 (moderate perinatal asphyxia) and 1.9% had an Apgar score under 3 (severe perinatal asphyxia) (Figure 3).

Resuscitation at birth was not required in IVF-LPT infants, in contrast to the non-IVF-LPT infants who required resuscitation (intermittent positive pressure ventilation by facial mask or Neo Puff) at 10.9%.

**Figure 4:**
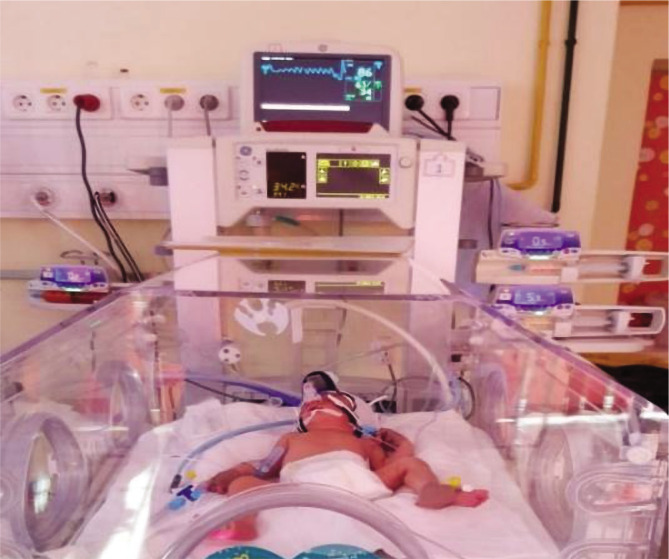
Late preterm newborn with O2 therapy – non-invasive ventilation.

Respiratory pathology in LPT remains at higher risk when compared with term infants [8]. This includes respiratory distress syndrome (RDS), transient tachypnea of the newborn (TTN), pneumonia, apnea, pulmonary hypertension, and pneumothorax (PTX). Speciality literature data show that RDS is still the most common respiratory disorder affecting late preterm infants, with an incidence of 5.2%–6.4%, decreasing from 10.5% at 34 weeks to 2.8% at 36 weeks. In our study, none of the IVF-LPT infants developed RDS, compared with spontaneous LPT newborns who developed RDS in a percentage of 5.9%. Transient tachypnea of the newborn was present in 18.1% of IVF-LPT newborns and 33.6% of non-IVF newborns (Table 1). Late preterm newborns conceived by *in vitro* fertilization, according to the results of studies in other countries, are more likely to require respiratory support, require more aggressive respiratory support and receive surfactant indicative of true respiratory distress syndrome [9]. In our study, IVF-LPT newborns required oxygen tent therapy in a smaller percentage than non-IVF LPT newborns, who required oxygen in 27.7% of cases and non-invasive mechanical ventilation-nasal (Figure 4) continuous positive airway pressure (CPAP) in 11.8% of cases (Table 1).

**Table 1:** Respiratory pathology and oxygen therapy in IVF and non-IVF-LPT infants.

Respiratory pathology	No IVF NB/%	Non-IVF.NB/%
**RDS**	(0%)	6 (5.9%)
**TTN**	2 (18.1%)	34 (33.6%)
**PTX**	(0%)	(0%)
**Apnea**	(0%)	(0%)
**O_2_ therapy**	**No IVF NB/%**	**Non-IVF.NB/%**
**Oxygen tent >24h**	28 (27.7%)	2 (18.1%)
**Non-invasive mechanical ventilation-nasal CPAP**	12 (11.8%)	-(0%)
**Invasive mechanical ventilation**	-(0%)	-(0%)

The low incidence of respiratory pathology and required oxygen therapy in IVF-LPT newborns was probably due to the prophylactic corticoid therapy on 100% of the pregnant IVF patients and very well-supervised IVF pregnancies (Table 2).

**Table 2:** Delivery mode, prenatal dexamethasone and type of pregnancy in IVF and non-IVF-LPT infants.

	No. IVF/%	No. Non-IVF/%
**Delivery mode/C-section**	11 (100%)	45 (44.55)
**RDS prevention/Prenatal Dexamethasone**	11 (100%)	5 (4.9%)
**Type of pregnancy/Multiple pregnancy**	10 (90.9%)	6 (5.9%)
**Supervised pregnancy**	11 (100%)	84 (83.1%)

Cesarean section was performed in a maximum percentage, probably because of the high IVF-LPT incidence of twins -90.9% *versus* 5.9% in the non-IVF group (Table 2) and to the desire for a successful pregnancy completion, subjectively arising, knowing that IVF techniques involve therapy stress, lack of certainty of success, high costs, time and energy.

Literature data show that IVF infants had a two-fold increase in odds of neonatal intensive care admission compared to spontaneously conceived newborns [10]. In our study, 45.4% of IVF-LPT infants required admission to NICU, but none more than 3 days, compared with non-IVF infants who required hospitalization in NICU for more than 3 days -19.8% (Table 3).

**Table 3:** Hospitalization in IVF and non-IVF-LPT infants.

		IVF-LPT		Non-IVF-LPT	
		No	%	No	%
**Hospitalization in NICU**	1–3 days	5	45.4%	36	35.6%
	>3 days	-	0%	20	19.8%
**Days of hospitalization**	<4 days	2	18.1%	19	18.8%
	4–7 days	7	63.8%	49	48.5%
	>7 days	2	18.1%	33	32.6%

The average hospitalization, between 4–7 days, is shorter than that of naturally conceived infants of the same age. This can be due to unsupervised pregnancies, underweight infants (less than 1500g), resuscitation at birth, and being prone to RDS.

## Discussion

IVF late preterm infants have an increased risk for respiratory morbidity, inversely proportional to gestational age. LPT newborns are at increased risk for respiratory distress syndrome (RDS), transitory tachypnea of the newborn (TTN), apnea, pulmonary hypertension (PPHN) and pneumothorax compared with newborns delivered at term [9].

Even if concerns have been raised over the past years, IVF has been used more frequently in recent years. Further research is needed to effectively evaluate IVF newborns and their risk.

## Conclusion

In our study, respiratory distress syndrome rarely occurred in late preterm IVF infants due to prenatal prophylactic treatment with corticosteroids. Respiratory pathology is rarely present due to careful monitoring during pregnancy, the presence of the neonatal team in the delivery room for possible resuscitation and providing proper care during the short hospitalization.
